# Comparison between 2D radiographic weight-bearing joint space width and 3D MRI non-weight-bearing cartilage thickness measures in the knee using non-weight-bearing 2D and 3D CT as an intermediary

**DOI:** 10.1177/20406223211037868

**Published:** 2021-08-21

**Authors:** Mylène P. Jansen, Simon C. Mastbergen, Felix Eckstein, Ronald J. van Heerwaarden, Sander Spruijt, Floris P. J. G. Lafeber

**Affiliations:** Department of Rheumatology and Clinical Immunology, University Medical Center Utrecht, Heidelberglaan 100 (G02.228), Utrecht 3584CX, The Netherlands; Department of Rheumatology & Clinical Immunology, University Medical Center Utrecht, Utrecht, The Netherlands; Department of Imaging and Functional Musculoskeletal Research, Institute of Anatomy and Cell Biology, Paracelsus Medical University Salzburg and Nuremberg, Salzburg, Austria; Ludwig Boltzmann Institute for Arthritis and Rehabilitation, Paracelsus Medical University, Salzburg, Austria Chondrometrics GmbH, Ainring, Germany; Centre for Deformity Correction and Joint Preserving Surgery, Kliniek ViaSana, Mill, The Netherlands; Reinier Haga Orthopaedic Centre, Zoetermeer, The Netherlands; Department of Rheumatology & Clinical Immunology, University Medical Center Utrecht, Utrecht, The Netherlands

**Keywords:** radiography, joint space width, MRI, cartilage thickness, correlation, CT, weight-bearing, 3D

## Abstract

**Background:**

In knee osteoarthritis, radiographic joint space width (JSW) is frequently used as a surrogate marker for cartilage thickness; however, longitudinal changes in radiographic JSW have shown poor correlations with those of magnetic resonance imaging (MRI) cartilage thickness. There are fundamental differences between the techniques: radiographic JSW represents two-dimensional (2D), weight-bearing, bone-to-bone distance, while on MRI three-dimensional (3D) non-weight-bearing cartilage thickness is measured. In this exploratory study, computed tomography (CT) was included as a third technique, as it can measure bone-to-bone under non-weight-bearing conditions. The objective was to use CT to compare the impact of weight-bearing *versus* non-weight-bearing, as well as bone-to-bone JSW *versus* actual cartilage thickness, in the knee.

**Methods:**

Osteoarthritis patients (*n* = 20) who were treated with knee joint distraction were included. Weight-bearing radiographs, non-weight-bearing MRIs and CTs were acquired before and 2 years after treatment. The mean radiographic JSW and cartilage thickness of the most affected compartment were measured. From CT, the 3D median JSW was calculated and a 2D projectional image was rendered, positioned similarly and measured identically to the radiograph. Pearson correlations between the techniques were derived, both cross-sectionally and longitudinally.

**Results:**

Fourteen patients could be analyzed. Cross-sectionally, all comparisons showed moderate to strong significant correlations (R = 0.43–0.81; all *p* < 0.05). Longitudinal changes over time were small; only the correlations between 2D CT and 3D CT (R = 0.65; *p* = 0.01) and 3D CT and MRI (R = 0.62; *p* = 0.02) were statistically significant.

**Conclusion:**

The poor correlation between changes in radiographic JSW and MRI cartilage thickness appears primarily to result from the difference in weight-bearing, and less so from measuring bone-to-bone distance *versus* cartilage thickness.

## Introduction

Knee osteoarthritis (OA) is a degenerative joint disease that is characterized by, among other factors, articular cartilage degeneration and subsequent thinning.^1^ The gold standard for quantifying cartilage thinning has traditionally been measurements of the joint space width (JSW) on weight-bearing radiographs.^2^ The radiographic JSW provides a two-dimensional (2D) projectional estimate of the bone-to-bone distance and thus reflects, to a certain extent, articular cartilage thickness. Radiographic JSW is often required for evaluating the rate of cartilage degeneration/regeneration in clinical trials and, when managed well with a high degree of acquisition standardization, the reliability and reproducibility of JSW measurement techniques are considered to be high.^3–5^ Because knee radiographs are generally taken in a weight-bearing position, quality of the cartilage (with respect to deformability of the tissue) may be an important factor in the assessment of radiographic JSW. However, representing only an indirect measure for cartilage thickness, JSW measurements can be influenced significantly by positioning, acquisition errors, focal cartilage degeneration, and changes in other joint tissues.^6,7^ The meniscus, in particular, has been shown substantially to impact radiographic JSW measurements.^8,9^

A more recent method is the direct measurement of articular cartilage thickness on magnetic resonance imaging (MRI) scans. Using MRI, cartilage tissue itself can be visualized three-dimensionally. Different quantitative measurements have been described and the average cartilage thickness generally shows high reproducibility.^10,11^ However, unlike radiography, MRI images are taken in a non-weight-bearing position. As such, deformability of the cartilage tissue is not taken into account. Yet, it has been shown that knee OA affects the mechanical properties of cartilage, which influences the amount of deformation.^12^

The literature comparing both techniques for natural OA progression shows moderate to strong correlations cross-sectionally.^13–15^ In cross-sectional evaluation, differences in cartilage thickness between individuals are relatively large (millimeters) and as such in favor of finding these relations. However, when looking at longitudinal changes over time, changes are much less pronounced (tenths of millimeters), limiting the measurement window. In these longitudinal studies, no or at best weak correlations were found between the change in radiographic JSW and the change in MRI cartilage thickness.^16–20^ This may be the result of the various differences between the techniques described previously: weight-bearing *versus* non-weight-bearing, bone-to-bone distance *versus* cartilage thickness, and 2D *versus* three dimensional (3D). In the present study we include CT as an imaging technique, as it is performed without weight-bearing, like MRI, but specifically visualizes the bone-to-bone distance, like radiographs. CT is a 3D imaging technique, but is also capable of creating a projectional image for 2D measurements. By including CT in the comparison with radiographic JSW and MRI cartilage thickness, the impact of weight-bearing *versus* non-weight-bearing and of measuring bone-to-bone JSW *versus* cartilage thickness measurements can be elucidated.

## Methods

### Patients

Patients treated with a joint-preserving surgical technique demonstrating cartilaginous tissue repair, knee joint distraction,^21–23^ who had radiographs (X-rays), MRI scans, and CT scans before and 2 years after treatment were included for this study. Knee joint distraction has previously been reported to result in cartilaginous tissue repair by radiographic and MRI evaluation, making it a population explicitly suitable for the present evaluation.^24^

Patients were included from two independent randomized controlled trials (RCTs).^25,26^ In both trials, a subgroup of patients (both *n* = 10) was asked to participate in an extended imaging protocol that included additional MRI and CT scans, in addition to the radiographs all patients received in these trials. The duration of patient follow-up was 2 years, and the images were originally used for evaluation of JSW (radiographs), cartilage thickness (MRI), and subchondral bone (CT) changes over time.^22,27,28^ The same images (analyses) were used for the current, post-hoc analyses to compare weight-bearing JSW and non-weight-bearing MRI cartilage thickness in a paired manner. As such, this is an exploratory study without sample size calculation, as no data were available as estimates for a power calculation. All available patients with complete imaging datasets at baseline and 2-year follow-up were included in the current study, to maximize statistical power. Both RCTs were granted ethical approval by the medical ethical review committee of the University Medical Center Utrecht (protocol numbers 10/359/E and 11/072) and registered in The Netherlands Trial Register (trial numbers NL2761 and NL2680). All patients gave written informed consent.

Knee joint distraction is a surgical treatment for end-stage knee OA below 65 years of age to postpone the need for a knee prosthesis.^29^ Inclusion and exclusion criteria of the RCTs and treatment details have been described previously.^27,30^ Before treatment, the most affected knee joint compartment (MAC), medial or lateral, was determined for all patients.

### Imaging and measurement methods

**Figure 1.**
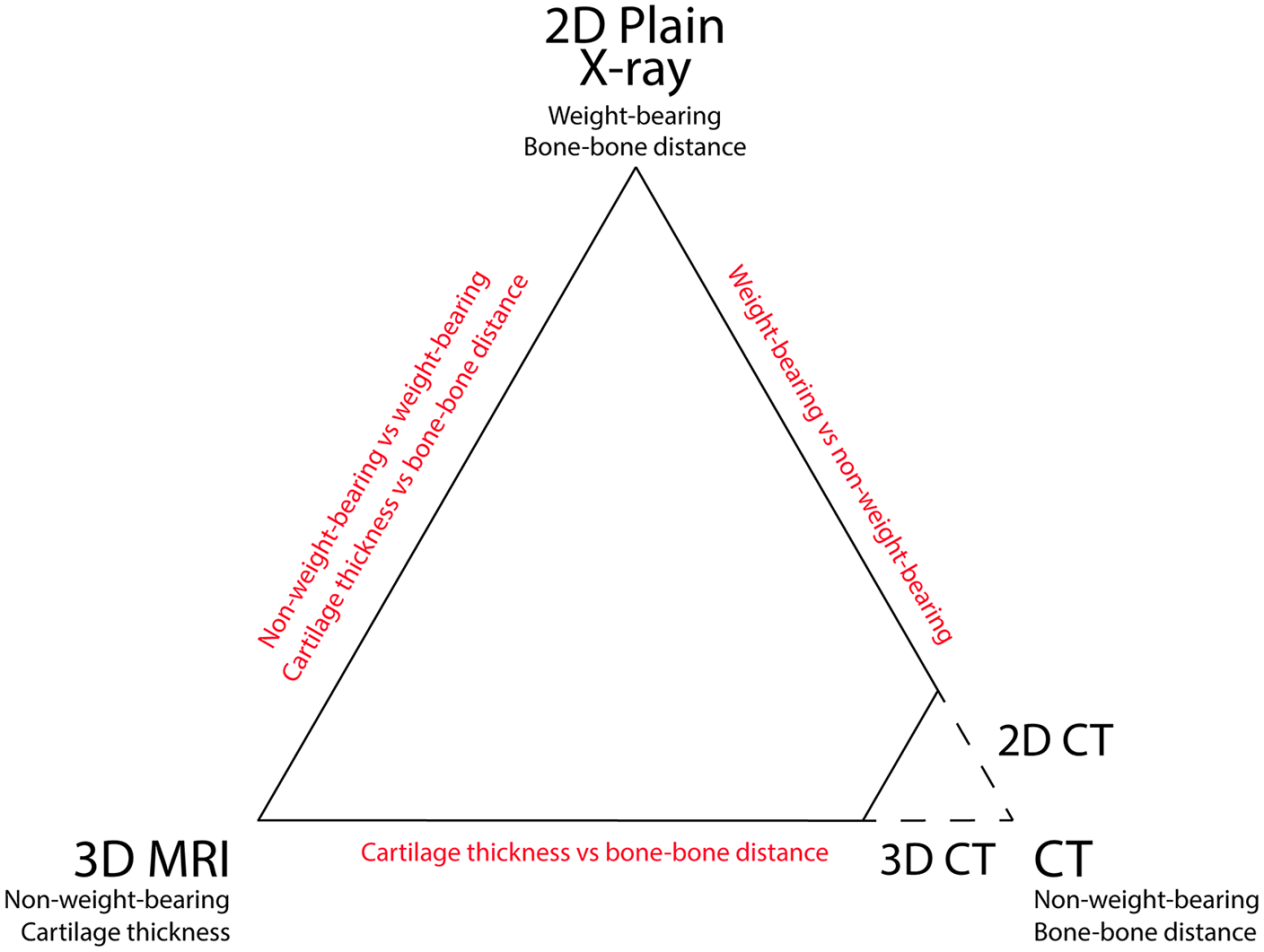
The three different imaging methods used for (in)direct cartilage quantification. The key characteristics are listed underneath each modality, and key differences between modalities are displayed in red. For computed tomography (CT), both three-dimensional (3D) and two-dimensional (2D) joint space width measurements were used, for comparison with magnetic resonance imaging (MRI) and radiography, respectively.

An overview of the different imaging techniques and key differences between them is shown in Figure 1.

#### Radiography (X-rays)

Standardized weight-bearing, semi-flexed, posteroanterior (PA) radiographs were performed according to the Buckland–Wright protocol.^31,32^ An aluminum step wedge was used as a reference standard to calculate the pixel size. For analysis of the radiographs, ‘knee images digital analysis’ (KIDA) software was used by one experienced observer, blinded to the acquisition order. The mean JSW of the MAC was calculated by averaging the tibia–femur distance at four locations of the MAC, which were determined automatically based on a framework of four lines placed manually around the joint. A detailed explanation of the KIDA mathematical method has been provided in the original article.^33^

#### Magnetic resonance imaging

3T MRIs with 3D spoiled gradient recalled imaging sequence with fat suppression (SPGR-fs) were acquired for analysis of cartilage structure using Chondrometrics Works 3.0 software.^34^ Experienced observers blinded to acquisition order segmented the tibiofemoral cartilage throughout the joint, which was averaged to calculate the mean cartilage thickness of the MAC.

#### Computed tomography

Axial CT scans of the knee were performed, from which coronal reconstructions with 2 mm slice thickness were rendered. A segmentation and 3D JSW measurement method was developed in-house. Bone segmentation was performed semi-automatically, after which the perpendicular distance from the tibia plateau to the femur was measured throughout the entire joint. Only tibial areas where the perpendiculars were ‘reflected’ back onto the tibia surface (i.e. the femoral perpendicular originating from the location where the tibial perpendicular meets the femoral surface has to meet the tibial surface as well) were included, to only include joint space areas where mutual force transfer between the two bones can take place. The medial and lateral boundaries were determined similar as for KIDA evaluation: the width of the medial and lateral sides of the joint are 3/20 of the total width of the joint, and the outer border of both sides is 2/15 of the total joint width away from the outer border of the joint, the latter was performed manually (MJ).^33^ The median of the remaining perpendicular distances of the MAC was calculated to get the ‘3D CT’ surface median JSW value. The median value instead of the mean value was used to exclude the influence of potentially artificially induced exceptionally large bone–bone distances; however, outcome was almost identical in case mean values were used.

In addition to the bone-to-bone distance of the 3D image, the coronal CT scans were rotated semi-automatically to a standard position in order to match the position used for the (weight-bearing) radiographs. The tibia plateau was positioned parallel to the axial plane and the line through the back of the femoral condyles was positioned parallel to the coronal plane, viz. the most optimal 2D image acquisition. The positioning of the tibia in relation to the femur was not changed (i.e. no artificial changes were made in the amount of flexion). Subsequently, an over-projection of the repositioned CT scan was created in the coronal plane, so that a non-weight-bearing 2D radiograph was mimicked. A wedge was added based on the current pixel size. These radiographs were then analyzed using the KIDA software, according to the same method and by the same observer as used for the weight-bearing radiographs. The ‘2D CT’ MAC mean JSW was calculated.

**Figure 2.**
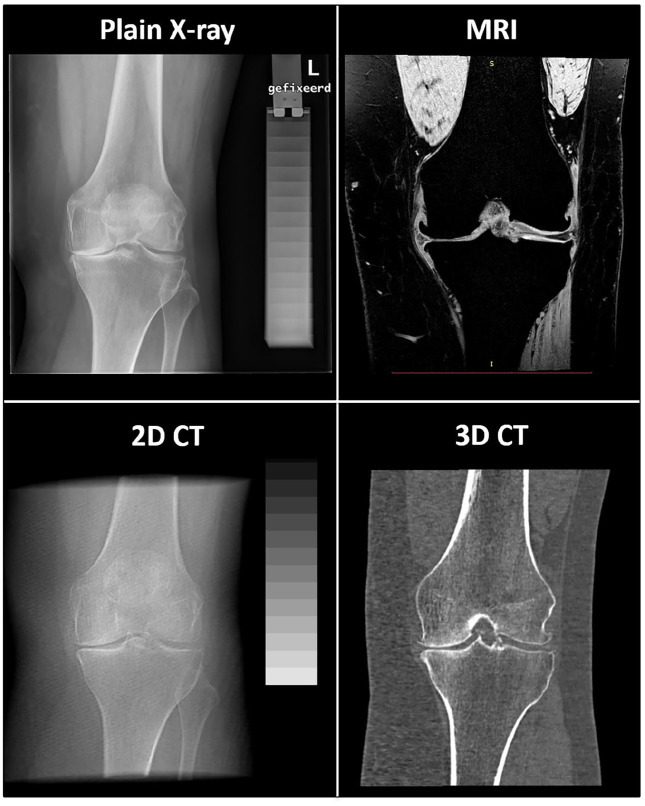
Representative image of the four techniques that are compared; all images are taken from the same patient before treatment (baseline). For magnetic resonance imaging (MRI) and three-dimensional (3D) computed tomography (CT) one slice is shown, as they are 3D imaging techniques. The two-dimensional (2D) CT images are created by over-projecting the CT scan, after standardized positioning, in the coronal plane.

A representative image of the four different techniques for the same patient is shown in Figure 2.

### Statistical analyses

For patient characteristics and image analysis results, descriptive statistics were used.

Pearson *R* correlations were calculated between the techniques cross-sectionally, using all patient time points in one comparison. In addition, Pearson *R* correlations between the techniques were calculated for the changes over time (2 years–baseline). To describe correlation strength, the guide for *R* values suggested by Evans in 1996 was used: <0.2 very weak; 0.2–0.39 weak; 0.40–0.59 moderate; 0.60–0.79 strong; >0.8 very strong.^35^ *p*-Values <0.05 were considered statistically significant. IBM SPSS Statistics version 25 (IBM Corp; Armonk, NY, USA) was used for all statistical analyses.

## Results

### Patients

Of the 20 patients originally included, three patients were lost to follow-up because they converted to a different treatment within 2 years after the original distraction treatment. Of one patient, no CT scan at baseline and 2 years was available. Of the remaining 16 patients, two had severe motion artefacts present in either of their two MRI scans disqualifying proper analyses. As such, 14 patients completed all imaging protocols at both time points and were used for evaluation.

The patient characteristics and image analysis results for the 14 included patients are shown in Table 1. Baseline parameters are comparable to those of the entire population of KJD patients from both original RCTs, as published before, so this small subpopulation seems representable for the entire KJD population.^27^

**Table 1.** Patient characteristics and image analysis (most affected compartment) results.

Patient characteristics	All patients (*n* = 14)		
	Mean ± SD or *n* (%)		
	Baseline		
Age, years	53.9 ± 7.7		
Weight, kg	87.6 ± 13.7		
BMI, kg/m^2^	27.6 ± 3.9		
Male gender	9 (64)		
Image analysis results	Baseline	2 Years	Δ2-year
X-ray JSW, mm	1.7 ± 1.9	2.7 ± 1.6	1.1 ± 1.3
MRI cartilage thickness, mm	2.0 ± 0.9	2.2 ± 0.9	0.2 ± 0.3
3D CT JSW, mm	4.4 ± 1.0	4.6 ± 0.8	0.2 ± 0.8
2D CT JSW, mm	4.2 ± 1.5	4.2 ± 1.5	0.0 ± 1.6

BMI, body mass index; CT, computed tomography; JSW, joint space width; KIDA, knee images digital analysis; MRI, magnetic resonance imaging; SD, standard deviation; 2D, two-dimensional; 3D, three-dimensional.

### Correlations

**Figure 3.**
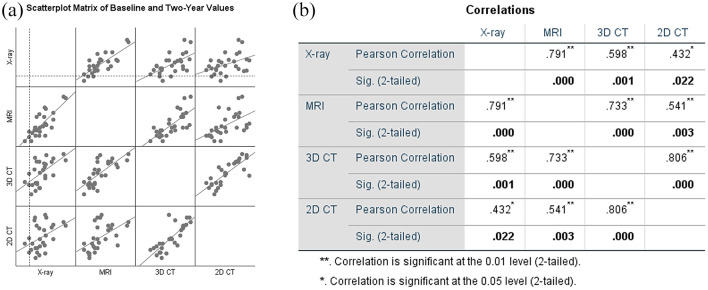
Cross-sectional correlations of combined baseline and 2-year values for all four techniques, displayed visually as a scatterplot matrix (a) and with Pearson *R* and *p*-values (b). The dotted line in (a) indicates the origin (0).

The cross-sectional correlations between all four techniques, of the baseline and 2-year values combined, are shown in Figure 3. The scatterplot matrix (left panel) shows that correlations were present between all techniques, as confirmed by the Pearson *R* and *p*-values (right panel). All correlations were statistically significant (all *p* < 0.023) and most were moderate or strong, with 2D CT and 3D CT showing a very strong correlation.

**Figure 4.**
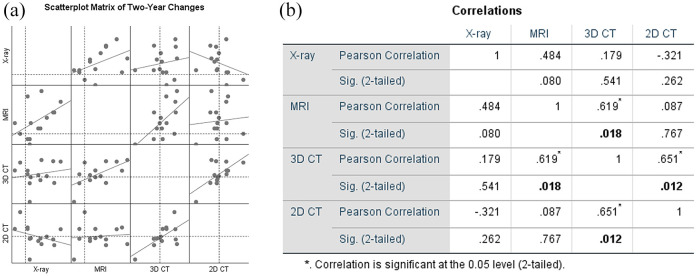
Correlations of 2-year changes over time for all four techniques, displayed visually as a scatterplot matrix (a) and with Pearson *R* and *p*-values (b). The dotted line indicates the origin (0).

The correlations between the 2-year changes of all four techniques are shown in Figure 4. It can be seen in the scatterplot matrix that between most techniques a clear correlation was absent. This was confirmed by the Pearson *R* and *p*-values.

**Figure 5.**
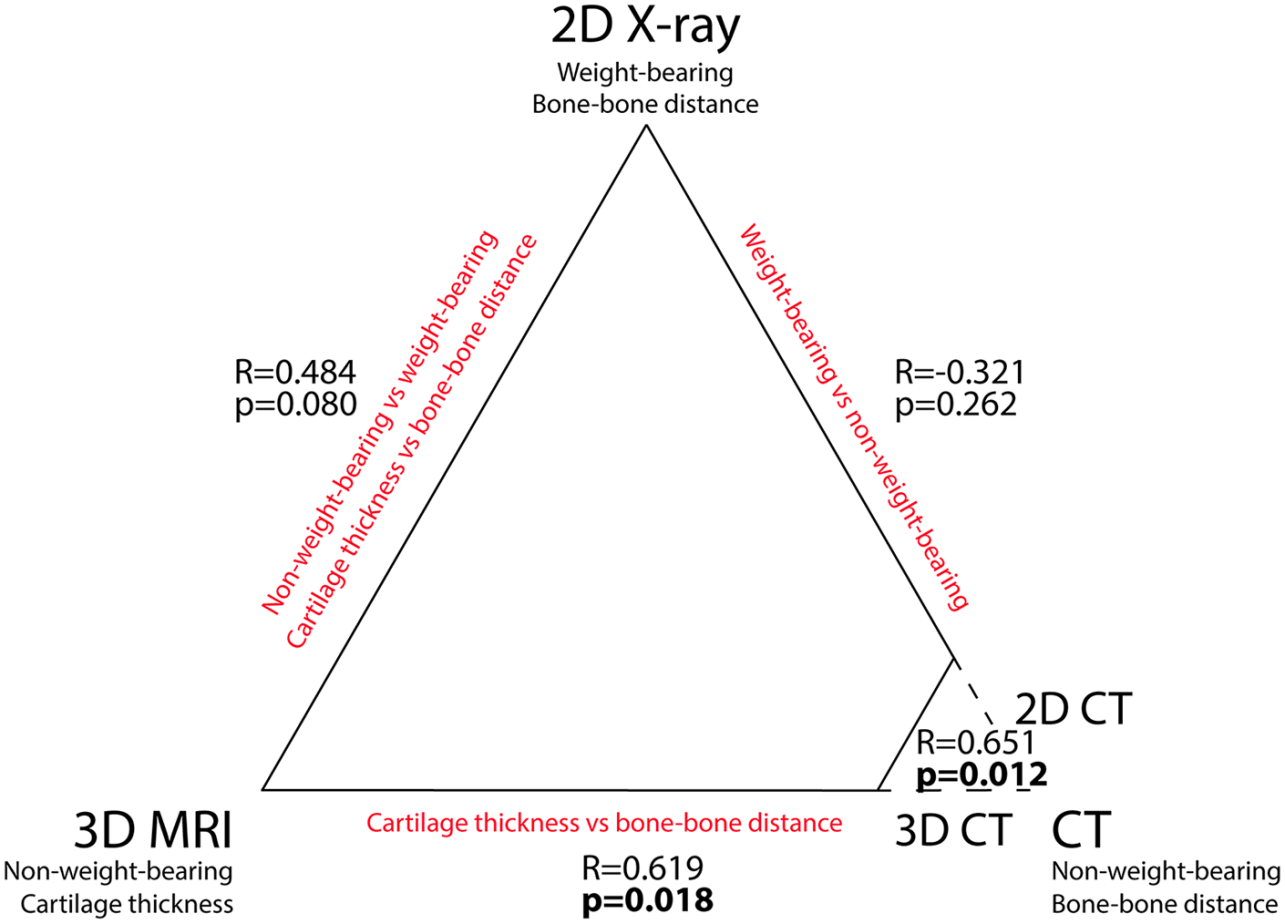
The three different imaging methods used for (in)direct cartilage quantification. The key characteristics are listed underneath each modality, and key differences between modalities are displayed in red. For computed tomography (CT), both three-dimensional (3D) and two-dimensional (2D) joint space width measurements were used, for comparison with magnetic resonance imaging (MRI) and radiography, respectively. Correlations (Pearson *R* and *p*-values) of the 2-year changes are shown between the techniques.

The change in radiographic (plain X-ray) mean JSW was not statistically significantly correlated with any of the other techniques, including the change in 2D CT JSW (Δ2D CT; correlation *R* = −0.321 and *p* = 0.262) and the change in MRI cartilage thickness (ΔMRI; correlation *R* = 0.484 and *p* = 0.080). There was a statistically significant, strong correlation between the change in 3D CT median JSW (Δ3D CT) and Δ2D CT mean JSW (*R* = 0.651; *p* = 0.012) and between Δ3D CT JSW and ΔMRI cartilage mean thickness (*R* = 0.619; *p* = 0.018). None of the other correlations were statistically significant. In Figure 5 these Pearson *R* and *p*-values have been added to the triangle of imaging techniques as depicted in Figure 1.

## Discussion

Although cross-sectional evaluation provided a statistically significant correlation between plain radiographic mean JSW (bone-to-bone distance) and MRI surface mean cartilage thickness, no statistically significant correlation between these measures was found when evaluating the relatively small changes over 2 years’ follow-up. Similarly, there was no significant correlation between the 2-year change in plain radiographic mean JSW and 2D CT mean JSW, whereas cross-sectional evaluation provided such a correlation. In contrast, the 2-year change in MRI surface mean cartilage thickness correlated strongly with 3D CT surface median bone-to-bone distance. Also, the 3D CT surface median JSW correlated strongly with the 2D mean JSW.

From this it is concluded that non-weight-bearing image acquisitions, independent of using evaluation of bone-to-bone distance measurements (CT) or cartilage thickness measurements (MRI), result in significant correlations between outcomes. In contrast, when a weight-bearing imaging technique (plain radiography) is compared to non-weight-bearing imaging techniques (MRI and CT) the correlation is lacking. It can therefore be concluded that weight-bearing image acquisition provides an independent characteristic of cartilage that is not observed by non-weight-bearing techniques. De-formability of the cartilage (cartilage quality) may be involved in addition to the quantitative measurement of cartilage thickness. The position and morphology of the meniscus may also play a role, although visually scored meniscal extrusion (grade 0–3) did not seem to influence the longitudinal correlation in this group of patients (data not shown) significantly.

The significant correlations found between the different imaging techniques when evaluating cross-sectional data, whereas such correlations are lost in the case of relating more subtle changes in cartilage quantitative measures during (2-year) follow-up, fits the inconclusive literature on this topic.^13–20^

With the exception of radiographic JSW, the 2-year changes over time in our study were much smaller than the absolute baseline or 2-year values (at least one order of magnitude decrease), while the standard deviations stayed roughly the same (Table 1). Apparently, correlations are lost when weight-bearing image acquisition is compared to non-weight-bearing acquisition in the case of small changes (over time), whereas they are maintained when bone-to-bone distance is compared to cartilage thickness in a 2D or 3D manner when the image acquisition is non-weight-bearing.

This argues for the use of weight-bearing image acquisition, such as weight-bearing CT or weight-bearing MRI. Both these techniques have been researched and have shown positive results, but the use of both is mostly limited to research settings.^36–38^ To investigate further the objectives of our study, a rotatable MRI scanner would be a valuable tool, because both cartilage thickness and JSW can be measured in weight-bearing and non-weight-bearing positions using the exact same imaging technique. Results of such future studies could help to relate better the results obtained from MRI scans and radiographs to monitor OA progression or treatment response. An important consideration in using weight-bearing CT or MRI is that using such approaches need thorough concern of the relative contribution of weight and cartilage deformability. Also, the actual weight-bearing relative to the contra-lateral leg in the case of uneven load distribution as well as pre-acquisition weight-bearing or exercise is a parameter to consider in such a study.^39^

A limitation of our study is the relatively small sample size, as only 14 of the original 20 complete datasets were available. As a sensitivity analysis, the two patients that were excluded because of MRI motion artefacts were included in the evaluation of radiographic JSW, 2D CT JSW and 3D CT JSW. The significance of the correlations between these three techniques for these 16 patients did not change compared to the (for all images complete) dataset of 14 patients, neither for absolute (cross-sectional) values nor for changes over time. Also, scatterplot matrices of all calculated correlations were included, because *p*-values may be less conclusive in this small number of patients. Clearly the scatterplot matrices support the conclusions based on the Pearson *R* and *p*-values. Irrespectively, the present study is a post-hoc analysis and is exploratory. Since no sample size calculation could be performed prior to analyses because no estimates were available, the achieved power was calculated after obtaining the data. For the cross-sectional comparison between MRI and JSW there was a power of 0.96 and 0.44 for the longitudinal comparison. For the longitudinal comparison between 3D JSW and MRI changes, the achieved power was 0.70. Clearly, the exploratory nature of the outcome of the study needs to be confirmed by larger datasets, and/or preferably using weight-bearing CT or MRI as additional variables. In particular, the latter would validate the conclusion.

Another limitation of our study is that knee flexion is not taken into account. The weight-bearing radiographs are performed under slight flexion of the knee (7–10°). MRI and CT scans are not performed under a specific angle, but normally the leg is extended for as much as is allowed by, for example, a patient’s possible extension limitation or the hardware set-up. Although the 3D imaging techniques provide a mean or median surface value, the 2D rendering of the 3D CT has a potential knee flexion angle difference as compared to the plain radiograph. This difference might have influenced the correlation between both techniques and the effect of different knee flexion could also be included in future research.

## Conclusion

In conclusion, the cause of the generally weak correlation between changes in radiographic JSW and MRI cartilage thickness appears primarily to be the difference in weight-bearing conditions during imaging, and less so the difference in measuring bone-to-bone distance *versus* cartilage thickness directly. Further research on the effects of weight-bearing on cartilage thickness measurements is warranted and might provide an indirect measure for cartilage deformability in the case of quantitative measurements, in addition to the measured thickness.
